# Risk factor for gametocyte carriage and gametocytemia in *Plasmodium vivax* and *Plasmodium falciparum*

**DOI:** 10.1186/s40249-025-01352-2

**Published:** 2025-08-04

**Authors:** Minxi Li, Yang Bian, Shishao Ruan, Zifang Wu, Di Zhang, Tongyu Ma, Yaming Wu, Xiao Liu, Duo Wang, Jia Lin, Danni Pan, Wenyan Cui, Lin Wang, Haichao Wei, Xuexing Zhang, Qinghui Wang, Weilin Zeng, Zhaoqing Yang, Yaming Cao, Liwang Cui, Daniel M. Parker, Yan Zhao

**Affiliations:** 1 Department of Immunology, College of Basic Medical Sciences, China Medical University, Shenyang, 110122 Liaoning China; 2 Department of Pathogen Biology and Immunology, Kunming Medical University, Kunming, China; 3 Division of Infectious Diseases and International Medicine, Department of Internal Medicine, Morsani College of Medicine, University of South Florida, Tampa, FL 33612 USA; 4 Department of Population Health and Disease Prevention, Department of Epidemiology & Biostatistics, University of California, Irvine, USA

**Keywords:** Gametocyte, *Plasmodium vivax*, *Plasmodium falciparum*, China-Myanmar border, Risk factor

## Abstract

**Background:**

Understanding *Plasmodium* sexual differentiation is crucial for blocking transmission. This study identified risk factors for gametocyte carriage and gametocytemia in *P. vivax* and *P. falciparum* to inform malaria elimination strategies at the China-Myanmar border.

**Methods:**

Gametocytes and asexual parasites were microscopically detected on thick smears collected from 2011 to 2020 in Laiza Township, Kachin State, Myanmar. Mono-/polyclonality were detected by genotyping at *Pvmsp3α*/*β* for *P. vivax*, and *Pfmsp1*/*2* for *P. falciparum*. Kulldorff’s retrospective time scan statistics tested for likely clusters of gametocyte-positive cases over time. Chi-square or Fisher’s exact tests compared proportions of gametocyte-positive cases in categorical variables. Generalized linear models assessed risk factors (year, season, demographics, clinical/parasitological features) for gametocyte carriage (logistic regression for a binomial outcome) and gametocytemia (Gaussian regression for continuous outcome), respectively.

**Results:**

During 2011–2020, 8240 patients had *P. vivax* infections, with 7249 testing positive for gametocytes. Among 510 *P. falciparum* cases, 56 tested positive for gametocytes. A significant cluster of *P. vivax* gametocyte carriage occurred from May 2015 to August 2017 (*P* = 0.001). For *P. vivax*, dry season, previous malaria history, fever, and parasite density were associated with gametocyte carriage. Gametocyte density increased with asexual parasite density (*P* < 0.001) but was lower during the rainy season and in those with a history of malaria infection (*P* < 0.001). Over time, gametocytes carriage proportion increased while density decreased (*P* < 0.001). For *P. falciparum*, younger age and previous malaria history were associated with gametocyte carriage, and density was higher in the dry season (*P* = 0.0115). Polyclonal *P. vivax* infections had higher gametocyte densities than monoclonal infections (*P* < 0.0001) and *P. falciparum* gametocyte density tended to increase with multiplicity of infection.

**Conclusions:**

Younger age, prior malaria infection, travel, and polyclonal infections correlate with higher *P. vivax* gametocyte prevalence. Gametocyte carriage peakes during the dry season, highlighting the need for seasonal strategies to support malaria elimination. These findings enhance understanding of risk factors for the transmissible stage of the two main human *Plasmodium* species in the Greater Mekong Subregion border areas.

**Graphical Abstract:**

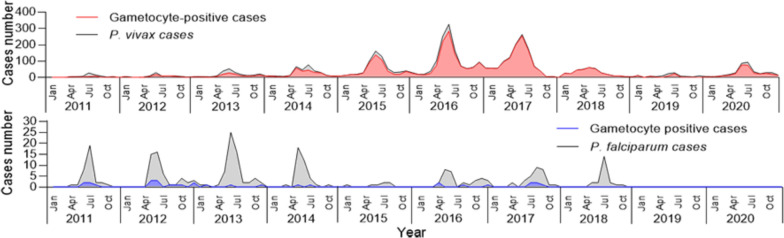

## Background

Malaria, an anopheline mosquito-transmitted infectious disease with *Plasmodium* parasites as the pathogen, poses a serious challenge to global public health [1, 2]. The latest *World Malaria Report* states that there were an estimated 263 million cases of malaria and 597,000 deaths in 2023, an increase of 11 million cases compared to 2022 [3]. With increasing resistance to antimalarial drugs and insecticides, and the coronavirus disease 2019 (COVID-19) pandemic disrupting many health efforts, the public health gains from malaria efforts have stalled or even reversed. Six countries of the Greater Mekong Subregion (GMS) of Southeast Asia have set targets to eliminate malaria by 2030. However, political turmoil and conflict (especially in Myanmar) have disrupted public health efforts and led to population movement from inland malaria endemic areas to remote border areas, where people live in poverty and public health infrastructure is poor, further exacerbating the problem of sustained malaria transmission along international borders [4]. Although there are no indigenous malaria cases in Chinese mainland, increasing commercial, academic, and personnel exchanges with malaria-endemic regions such as Southeast Asia and Africa raise the risk of reintroduction. This population movement and connectivity between malarious and malaria-receptive areas (such as parts of China where malaria vectors continue to exist) make it possible for malaria to re-spread in China [5].

The human-to-mosquito transmission of malaria parasites completely depends on the sexual gametocyte stage [6]. *Plasmodium falciparum* gametocytes appear in peripheral blood approximately 7–15 days after the initial erythrocytic cycle, whereas those of other species (e.g. *Plasmodium vivax*) appear much earlier, within 1–3 days [7–9]. Unlike other species, *P. vivax* can form hypnozoites in hepatocytes, and its gametocytes develop well before the parasites enter the bloodstream. It has been suggested that the gametocytes of *P. vivax* are derived from merozoites in the liver stage, which could explain why patients infected with *P. vivax* can transmit without showing clinical symptoms [10–12]. The factors associated with gametocyte carriage are complex, and the mechanisms are not yet fully understood. Human genetic background, environmental factors (including climatic and meteorological factors), asexual parasitemia, allelic differences in *Plasmodium* strain-specific genes, and host fever or hematopoiesis may all affect sexual differentiation, sexual orientation, gametocyte maturation and viability, and ultimately transmission to mosquitoes [13, 14].

Multiplicity of infection (MOI), the number of different parasite genotypes that co-exist in a given infection, is considered a meaningful metric for describing the intensity of malaria transmission (with MOI normally thought to be higher in settings with higher transmission) [15]. In *P. falciparum* infections, merozoite surface protein 1 (*Pfmsp1*) and merozoite surface protein 2 (*Pfmsp2*) are commonly used as polymorphic markers to distinguish genetic variation in the parasite. Differentiating parasite genotypes in a specific infection from 1 (monoclonal infection) to 10 (polyclonal infection) can be distinguished using this method in endemic settings [16, 17]. For *P. vivax*, merozoite surface protein 3α (*Pvmsp-3α*) and merozoite surface protein 3β (*Pvmsp-3β*) genotyping are often used to identify the genetic diversity of the population. A positive correlation has been observed between MOI and gametocyte carriage, which in turn is associated with increased mosquito infection rates [18, 19].

Heterogeneity in the transmission of malaria has been documented at multiple scales. For instance, geographic clusters with high malaria burdens are frequently observed in many malarious regions, and these clusters can act as reservoirs for feeding persistent or recurrent malaria cases in other regions through human and mosquito movement. At more micro-scales, heterogeneities have been documented in individual susceptibility to malaria infection, exposure to mosquito vectors, and infectiousness [20–23]. In this study, we implemented passive case surveillance at a treatment facility in Laiza Township, Kachin State, Myanmar. Using data from this malarious border region we investigated parasite and host-related factors associated with gametocyte carriage and explored the extent to which specific *Plasmodium* variants are linked to gametocyte carriage. The results of these analyses are relevant for understanding factors influencing the natural formation of malaria gametocytes in infections, for understanding heterogeneities in potential infectiousness, and potentially for developing localized control measures aimed at preventing malaria transmission along the China-Myanmar border areas.

## Methods

### Study site and sample collection

The research site is located in Laiza Township (24°45′N, 97°33′E, elevation 263 m), northeast of Myanmar, adjacent to Nabang Township, Yingjiang County, Yunnan Province, China. Since 2011, the malaria investigation team of the Southeast Asia International Center of Excellence for Malaria Research (ICEMR) established a local malaria monitoring station and implemented passive case detection (PCD) in Laiza Township. In 2010, following military conflict between the local and central governments of Myanmar, some internally displaced people settled about 5 km from the Laiza town, establishing border refugee camps. From 2011 to 2017, we obtained information on malaria cases from Laiza Hospital and other clinics, including internally displaced person (IDP) camps. Since 2018, due to limited staffing resources, case data have been collected exclusively from Laiza hospital. To ensure consistency in data sources, malaria case trends from Laiza Hospital were analyzed over the period from 2011 to 2020. All available samples, including those from clinics serving the IDP camps, were used to analyze factors associated with gametocyte carriage and gametocytemia.

Patients with fever (defined as axillary temperature ≥ 37.5 °C) or suspected malaria patients with a history of fever within the past 24 h were included in the study. Demographic characteristics (ethnicity, age, sex, occupation, education level and travel history) and clinical data (body temperature, fever history, malaria history, previous treatment, use of mosquito nets, etc.) were collected using structured questionnaires. In order to determine the species and density of malaria parasites, peripheral blood was obtained through standard finger puncture methods to prepare thick and thin blood smears and dry blood spots which were stored at − 20 ℃ and used for later molecular detection experiments. All participants signed consent forms and were interviewed prior to sampling. This study was reviewed and approved by the Institutional Review Committee of the University of South Florida and China Medical University.

### Microscopic analyses

For malaria diagnosis, thick and thin smears were prepared from finger-prick blood samples collected from patients, stained with Giemsa, and examined under a light microscope (LM). Initial diagnosis was performed at local hospitals or clinics by field microscopists. Patients with confirmed malaria were treated by hospital and clinic staff according to the local malaria treatment guidelines (described in [24]). All blood smears were subsequently transported to a nearby project field laboratory and re-examined by two experienced microscopists to identify *Plasmodium* species and quantify parasite densities of both asexual and sexual stages. Slides were double-read under microscopy, with asexual parasite densities first assessed against 200 white blood cells (WBCs). When sexual forms (gametocytes) were observed, their density was determined against 500 WBCs. A third microscopist was consulted in cases of discrepancies, defined as follows: (i) difference in *Plasmodium* species identification; (ii) positive versus negative results; and (iii) if the higher count divided by the lower count was ≥ 2. In such cases, the two closest values among the three readings were used to calculate the final result. Parasite density (parasites/μl of blood) was calculated by dividing the number of parasites counted by the number of WBCs observed and multiplying the result by an assumed standard of 8000 WBCs/μl. If no parasites were observed after examining 100 high-power fields at 1000 × magnification [(100 × objective) × (10 × ocular)], the result was recorded as negative [25]. The final parasite density was calculated as the arithmetic mean of the two or three readings, and per 200 WBC for asexual stage parasites or per 500 WBC for sexual stage parasites. The microscopists involved in this study were certified by the Myanmar National Malaria Control Program and had ≥ 5 years of experience in malaria diagnosis in endemic settings.

### Molecular analyses

Genomic DNA (gDNA) was extracted from DBSs using the QIAamp DNA Minikit (Qiagen, Hilden, Germany) following manufacturer’s instructions. Genotyping methods for *Pvmsp-3α* and *Pvmsp-3β* have been previously described [26]. For the initial amplification, 1.0 μl of gDNA was added to a 10 μl reaction mixture containing 1.0 μl of 10 × PCR buffer, 0.8 μl of dNTPs, 0.2 μl of r*Taq* polymerase (Takara, Beijing, China), 0.2 μl of each primer (10 pmol/L), and distilled H₂O to reach the final volume. In nested PCR, 1.0 μl of the primary amplification product was used as the template in a total reaction volume of 10 μl. The PCR products were separated by electrophoresis on a 1.2% agarose gel and analyzed accordingly. Allelic polymorphism of the *Pvmsp-3α* and *Pvmsp-3β* loci were assessed using established polymerase chain reaction-restriction fragment length polymorphism (PCR–RFLP) techniques. Briefly, 4 μl of each PCR product was digested with restriction enzymes for one hour at 37 °C. The enzymes *Hha*I and *Pst*I (Takara, Beijing, China) were used to digest *Pvmsp-3α* and *Pvmsp-3β* amplicons, respectively, in separate reactions with a total volume of 20 μl. The resulting restriction fragments were resolved on a 1.2% agarose gel and visualized accordingly. Mixed-strain infections were inferred when multiple-sized PCR products appeared during initial amplification or when the combined size of RFLP-restricted DNA fragments exceeded that of the uncut PCR product.

Nested PCR was employed to genotype the *Pfmsp1* block 2 (K1, MAD20 and RO33) and *Pfmsp2* block 3 (3D7 and FC27) allelic families. Briefly, the first round of PCR was performed using 1 µl of gDNA extract. The cycling conditions for the primary PCR round included an initial denaturation step of 2 min at 94 °C, followed by 30 cycles of 30 s at 94 °C, 1 min at 45 °C and 2 min at 72 °C, and a final extension step of 5 min at 72 °C. The nested PCR was performed using 1 μl of the primary PCR product as template. For the nested *Pfmsp1 and Pfmsp2* rounds, the cycling conditions consisted of an initial denaturation step of 2 min at 94 °C followed by 35 cycles of 30 s at 94 °C, 1 min at 58 °C and 2 min at 72 °C, and a final extension step of 5 min at 72 °C. PCR amplicons were visualized following agarose gel electrophoresis using a GIS gel imaging system (UVP, Upland, USA). MOI was calculated separately for each locus, and the highest MOI per clinical isolate was used, as previously described in detail [16, 17].

### Temporal cluster analysis

We used Kulldorff’s retrospective temporal scan statistics to identify potential clusters of vivax gametocyte positive cases over time. Briefly, the process involves scanning temporal windows and comparing the observed and expected number of cases within each window to those outside the window. A likelihood ratio test was used to assess differences in malaria risk within and outside the scanning window, under the null hypothesis of equal risk over time. The window with the maximum log likelihood ratio (LLR) was considered the most likely cluster with the highest malaria risk [27]. Temporal clustering analysis was conducted for each year from 2011 to 2020, with a temporal aggregation of one month. Additionally, the data were also analyzed for the entire study period (2011–2020). Sscan statistics were computed using SaTScan™ version 9.3 (Kulldorff M and Information Management Services, Inc., https://www.satscan.org/).

### Seasonal fluctuation analysis

The seasonal index for each month was calculated by dividing the 10-year average number of cases in that month by the overall monthly mean across the entire study period, to assess seasonal variation. No substantial seasonal fluctuation would be expected if the monthly seasonal indices were all close to 1 [28].

### Statistical analysis

Chi-square tests or Fisher’s exact tests were used to compare proportions of gametocyte-positive cases across categorical variables. Generalized linear models (GLM) were used to investigate the effect of season, gender, age, ethnicity, malaria history, travel history, temperature, and parasite density on gametocyte carriage using a binomial distribution (i.e., a logistic regression). The same covariates were used in a GLM with a Gaussian distribution to identify predictors of gametocytemia (continuous outcome, with gametocytes per WBC). Adjusted odds ratios (a*OR*) were used to quantify the magnitude of association between the factors of interest and gametocyte carriage in the logistic regression. The Wilcoxon rank sum test was used for non-parametric analyses. Standard methods were used to compute 95% confidence intervals (*CI*), and *P* values were interpreted as two-tailed. *P* < 0.05 indicates a statistically significant difference. All statistical analysis were performed using SPSS Statistics version 24.0 (SPSS Inc., Chicago, USA) and R software version 4.3.1 (R Core Team, R Foundation for Statistical Computing, Vienna, Austria).

## Results

From January 2011 to January 2020, 46,547 questionnaires were collected from hospitals, clinics, and refugee camps. Among these, 8519 *P. vivax* and 542 *P. falciparum* cases were confirmed via microscopic examination. After excluding cases with incomplete information, a total of 8240 *P. vivax* and 510 *P. falciparum* cases were included in the risk factor analysis, with gametocyte positivity rates of 88.0% and 11.0%, respectively. Mixed infections of *P. vivax* and *P. falciparum* were excluded from the analysis due to the extremely low number of cases. Notably, Laiza Hospital was the only site that continuously collected samples throughout the entire study. From this hospital, 4580 *P. vivax* and 268 *P. falciparum* cases were identified, with gametocyte positivity rates of 86.1% and 11.2%, respectively. These cases were specifically included to analyze the trend of gametocyte positivity over time.

### Temporal patterns in gametocyte carriage

**Fig. 1:**
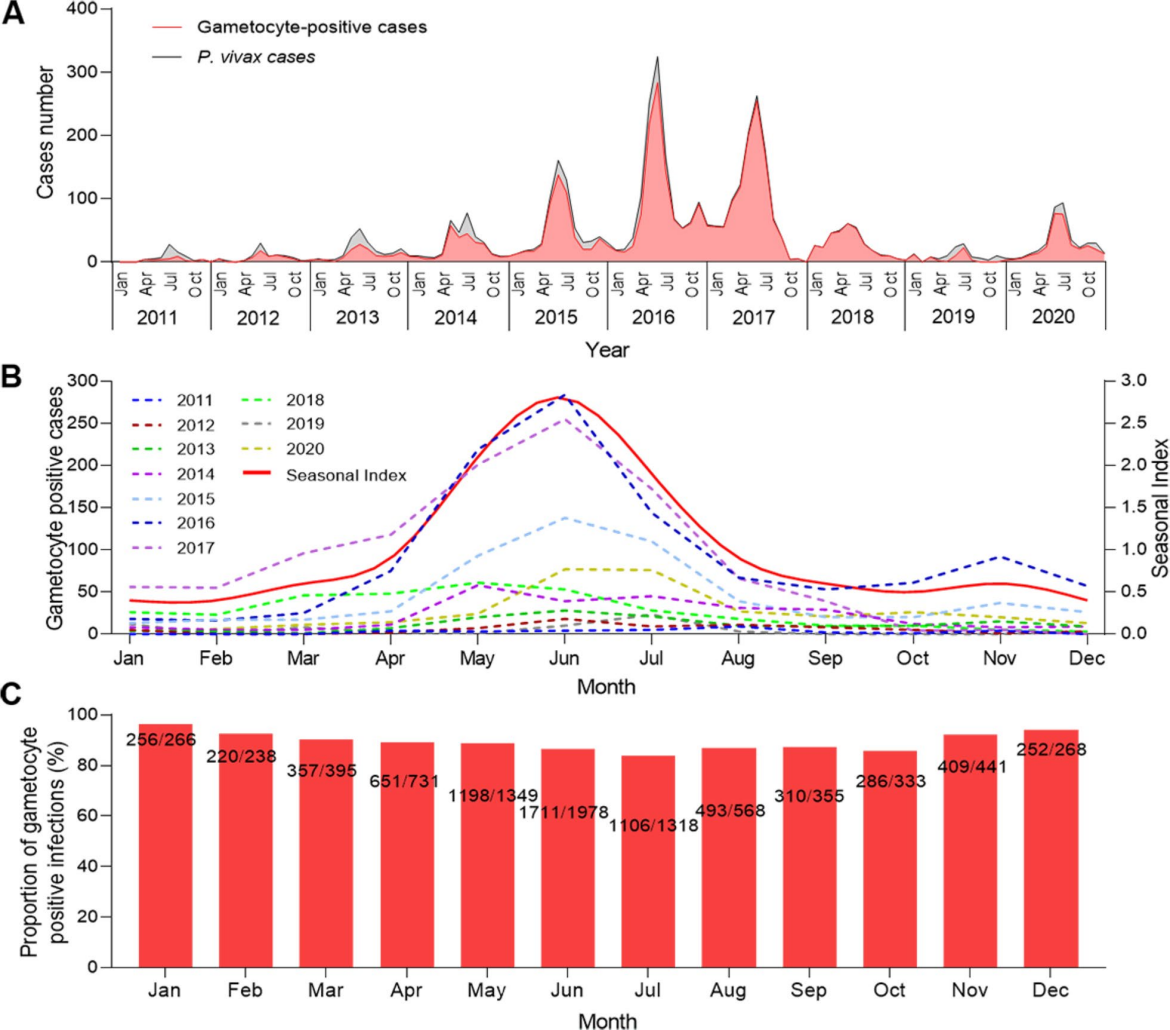
Trend and dynamics of gametocyte carriage of *Plasmodium vivax* in Laiza township at the China-Myanmar border during 2011–2020. **A** Temporal case counts of total *P. vivax* infections (black line) and gametocyte-positive cases (red line). **B** Monthly gametocyte positive cases in different years (left y-axis) and average seasonal index (right y-axis). **C** Monthly proportion of gametocyte positive cases

First, we identified a major outbreak of *P. vivax* during the 10-year study period which occurred between 2015 and 2017. Notably, there was a rebound in 2020 compared with the cases in 2019 (Fig. 1A). Additionally, the trend of gametocyte-positive *P. vivax* cases was consistent with the overall fluctuations in malaria cases from 2011 to 2020 (Fig. 1A). Gametocyte-positive cases exhibited a distinct seasonal pattern, with the majority occurring during the rainy season (May to October), and a peak observed from May to July (Fig. 1B). The seasonal gametocyte index reached its highest level in June [seasonal index (SI) = 2.8]. The proportion of gametocyte-positive infections ranged from 83.8% in July to 96.2% in January (Fig. 1C).

**Fig. 2:**
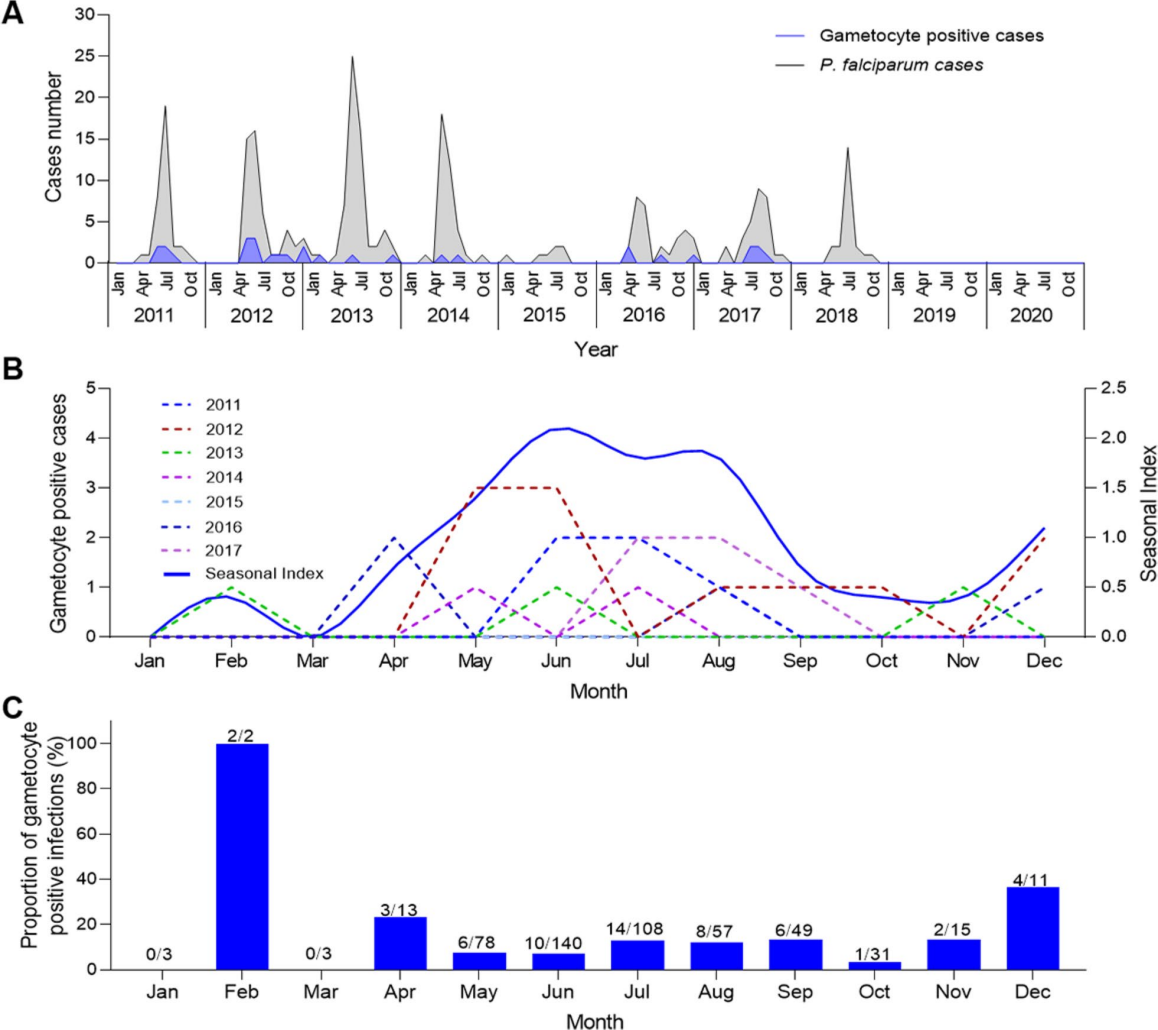
Trend and dynamics of gametocyte carriage of *Plasmodium falciparum* in Laiza township at the China-Myanmar border during 2011–2020. **A** Temporal case counts of total *P. falciparum* infections (black line) and gametocyte-positive cases (blue line). **B** Monthly gametocyte positive cases in different years (left y-axis) and average seasonal index (right y-axis). **C** Monthly proportion of gametocyte positive cases. No gametocyte-positive cases were detected by microscopy after 2017, and *P. falciparum* transmission ceased after 2018

There were relatively few cases of *P. falciparum* at the China-Myanmar border, with an overall downward trend in recent years. Notably, no cases of *P. falciparum* have been reported since 2018 (Fig. 2A). The seasonal trend showed that most cases still occurred during the rainy season, peaking from May to July (Fig. 2B). The seasonal index of gametocyte-positive cases peaked in June (SI = 2.1). Additionally, the proportion of gametocyte-positive infections was significantly higher during the dry season (November to April) than during the rainy season (May to October) (*P* = 0.0042, Chi-square test) (Fig. 2C).

The duration of temporal clusters of gametocyte-positive cases progressively shortened over the study period, from April–August 2011 to June–July 2020 (Table 1). Temporal cluster analysis revealed that gametocyte carriage mainly occurred during the rainy season, particularly in June to July. The specific months with higher temporal clustering exhibited minor variations across different years. A particularly significant temporal cluster of gametocyte carriage was observed between May 2015 and August 2017 in the study area (LLR = 1629.69, *P* = 0.001) (Table 1). During this period, a total of 2613 gametocyte-positive cases were recorded, and the relative risk of malaria infection was 6.43 compared to other periods (Table 1).

**Table 1:** Clusters of vivax gametocyte positive cases, detected using Kulldorff’s purely temporal clustering approach, in Laiza 2011–2020

Year	Cluster time frame^a^	Observed	Expected	RR	LLR	*P-*value
All years						
2011	2011/4 to 2011/8	25	13.41	4.95	8.73	0.001
2012	2012/5 to 2012/9	53	28.84	4.61	17.52	0.001
2013	2013/5 to 2013/7	69	34.53	3.01	19.88	0.001
2014	2014/5 to 2014/9	202	108.99	4.83	69.14	0.001
2015	2015/5 to 2015/7	341	140.39	4.68	160.73	0.001
2016	2016/5 to 2016/7	647	279.27	4.15	272.79	0.001
2017	2017/3 to 2017/7	842	447.26	5.19	304.71	0.001
2018	2018/3 to 2018/6	208	110.64	3.37	59.59	0.001
2019	2019/6 to 2019/7	33	10.36	5.67	21.49	0.001
2020	2020/6 to 2020/7	153	53.17	4.61	83.56	0.001
10 years						
2011–2020	2015/5 to 2017/8	2613	922.26	6.43	1629.69	0.001

*RR* relative risk; *LLR* log likelihood ratio

^a^Clusters are identified by comparing the observed number of cases within each window to the expected number under a random distribution, while scanning across varying time intervals

### Characteristics of gametocyte positivity among detected *P. vivax* and *P. falciparum* cases

All samples, including those collected from IDP camp clinics, were analyzed to identify factors associated with gametocyte carriage and gametocytemia. A total of 8240 patients with *P. vivax* infection were included in the study, of whom 7249 tested positive for gametocytes (Table 2). Regarding seasonal distribution, the proportion of vivax malaria cases with concurrent gametocyte carriage was significantly higher in the dry season than in the rainy season (91.7% vs 86.5%, *P* < 0.001). Among patients without a history of malaria, 86.4% were gametocyte-positive, which was significantly lower than the 98.1% observed among those with a reported history of malaria infection (*P* < 0.001). Regarding parasite density, the prevalence of gametocytes carriage was 91.4% in *P. vivax* infections with parasite densities > 500 parasites/μl, significantly higher than the 78.2% observed in infections with densities < 500 parasites/μl (*P* < 0.001) (Table 2). No statistically significant differences in gametocyte positivity were observed across categories of gender (*P* = 0.407), age (*P* = 0.088), ethnicity (*P* = 0.457), occupation (*P* = 0.062), education (*P* = 0.115), travel history (*P* = 0.295), and fever status (*P* = 0.962) (Table 2).

**Table 2:** Characteristics of gametocyte carrier cases in *Plasmodium vivax* and *P. falciparum*

Characteristics	*P. vivax*			*P. falciparum*		
	Total	Gamet. + , *n* (%)	*P-*value	Total	Gamet. + , *n* (%)	*P-*value
All	8240	7249		510	56	
Season			** < 0.001**			**0.004**
Dry	2339	2145 (91.7)		47	11 (23.4)	
Rainy (May to October)	5901	5104 (86.5)		463	45 (9.7)	
Sex			0.407			0.165
Male	4914	4311 (87.8)		380	46 (12.1)	
Female	3326	2938 (88.3)		130	10 (7.7)	
Age (years)			0.088			**0.002**
< 5	776	691 (89.0)		19	5 (26.3)	
5–14	2785	2473 (88.8)		92	16 (17.4)	
15–24	2782	2444 (87.9)		215	25 (11.6)	
25 +	1897	1641 (86.5)		184	10 (5.4)	
Ethnicity			0.457			0.761
Non-Kachin	267	231 (86.5)		30	2 (6.7)	
Kachin	7973	7018 (88.0)		480	54 (11.3)	
Occupation			0.062			**0.047**
Indoor worker^a^	1308	1134 (86.7)		76	7 (9.2)	
Manual labor^b^	345	301 (87.2)		-	-	
Farmer	1093	943 (86.3)		108	7 (6.5)	
Soldier	806	729 (90.4)		154	17 (11.0)	
Student	3804	3363 (88.4)		141	17 (12.1)	
Other^c^	884	779 (88.1)		23	8 (29.4)	
Education			0.115			0.338
Primary or less	3766	3312 (87.9)		177	24 (13.6)	
Middle/high school	4325	3814 (88.2)		319	30 (9.4)	
University above	149	123 (83.2)		14	2 (14.3)	
Malaria history^d^			** < 0.001**			** < 0.001**
No	7144	6174 (86.4)		464	23 (5.0)	
Yes	1096	1075 (98.1)		46	33 (71.7)	
Travel history			0.295			1.000
No		7199 (87.9)		492	54 (11.0)	
Yes		50 (92.6)		18	2 (11.1)	
Fever			0.962			0.083
No	2622	2306 (87.9)		117	18 (15.4)	
Yes	5618	4943 (88.0)		393	38 (9.7)	
Parasite density (parasites/μl)			** < 0.001**			0.743
≤ 500	2110	1649 (78.2)		90	9 (10.0)	
> 500	6130	5600 (91.4)		420	47 (11.2)	

^a^Indoor workers include office workers, housewives/housekeepers, and teachers

^b^Manual labor includes factory/construction workers, lumberjack, plantation workers, temporary job/labor, hunters, miners, herdsman and gardeners/bush clearing

^c^Other include non-school child and business person

*Gamet* Gametocyte

Bold indicates a *P* value less than 0.05

Among the 510 patients infected with *P. falciparum*, 56 were positive for gametocytes. Similar to *P. vivax* infections, significant seasonal variation in gametocyte carriage was observed (*P* = 0.004), along with a significant difference between individuals with and without a history of malaria infection (*P* < 0.001) (Table 2). The highest proportion of gametocyte carriers was observed in children under 5 years old (26.3%), followed by those aged 5‒14 years (17.4%). Gametocyte carriage rates differed significantly across age groups (*P* = 0.002). Regarding occupation, the highest gametocyte carriage rate was found in non-school age children and business persons (lumped together into a category named “Other”), at 29.4%, followed by students and soldiers (12.1% and 11.0% respectively); these differences were also statistically significant (*P* = 0.047) (Table 2). No statistically significant differences in gametocyte positivity were found based on gender (*P* = 0.165), ethnicity (*P* = 0.761), education (*P* = 0.338), travel history (*P* = 1.000), or fever status (*P* = 0.083) (Table 2).

### Results from logistic regression for risk factors for gametocyte carriage among vivax and falciparum malaria positive individuals

The results of the multivariable analysis revealed several statistically significant factors that influence gametocyte carriage in *P. vivax* infections. Firstly, seasonal factors were significantly associated with lower odds of gametocyte positivity during the rainy season compared to the dry season (a*OR* = 0.74, 95% *CI:* 0.62–0.89, *P* = 0.0013) (Table 3). Furthermore, patients with a history of malaria in the past 12 months had higher odds of gametocyte positivity compared to those without a previous malaria history (a*OR* = 17.54, 95% *CI:* 11.20–28.90, *P* < 0.001) (Table 3). Similarly, the density of *Plasmodium* parasites in the patients also had a statistically significant effect. Patients with high parasite density had higher odds of gametocyte positivity compared to those with low parasite density (a*OR* = 2.61, 95% *CI:* 2.31–2.95, *P* < 0.001) (Table 3). Compared to children under 5 years old, the odds of gametocyte carriage were lower in the 15–24 year age group (a*OR* = 0.75, 95% *CI*: 0.56–0.99, *P* = 0.0466) (Table 3). Body temperature (i.e., fever) was also associated with gametocyte carriage. Compared to patients with normal body temperature, those with fever had higher odds of gametocyte infection (a*OR* = 1.36, 95% *CI:* 1.15–1.61, *P* < 0.001) (Table 3). Additionally, there was a variation in *P. vivax* gametocyte carriage across different years (a*OR* = 2.01, 95% *CI:* 1.90–2.12, *P* < 0.001) (Table 3).

**Table 3:** Results from logistic regression for risk factors for gametocyte carriage in *Plasmodium vivax* and *P. falciparum* infections

Variables	*P. vivax*		*P. falciparum*	
	a*OR* (95% *CI*)	*P-*value	a*OR* (95% *CI*)	*P-*value
Season				
Dry	1		1	
Rainy (May to October)	0.74 (0.62–0.89)	**0.0013**	0.39 (0.14–1.20)	0.0881
Sex				
Male	1		1	
Female	1.00 (0.86–1.17)	0.9966	0.69 (0.25–1.70)	0.4315
Age (years)				
< 5	1		1	
5–14	0.96 (0.72–1.27)	0.7788	0.50 (0.10–2.79)	0.4051
15–24	0.75 (0.56–0.99)	**0.0466**	0.22 (0.05–1.19)	0.0678
25 +	0.87 (0.65–1.17)	0.3606	0.16 (0.03–0.85)	**0.0257**
Ethnicity				
Non-Kachin	1		1	
Kachin	0.95 (0.62–1.43)	0.8033	16.94 (0.96–674.02)	0.0885
Malaria history^a^				
No	1		1	
Yes	17.54 (11.20–28.90)	** < 0.001**	66.76 (27.17–180.31)	** < 0.001**
Travel history				
No	1		1	
Yes	1.89 (0.62–7.24)	0.2980	4.16 (0.33–25.45)	0.1786
Fever				
No	1		1	
Yes	1.36 (1.15–1.61)	** < 0.001**	0.62 (0.24–1.61)	0.3160
Parasite density (Log, parasites/μl)	2.61 (2.31–2.95)	** < 0.001**	0.85 (0.73–0.99)	**0.0358**
Year	2.01 (1.90–2.12)	** < 0.001**	1.16 (0.94–1.44)	0.1601

*aOR* adjusted odds ratios, *CI* confidence interval

^a^ Malaria reported in the past 12 months

Bold indicates a *P* value less than 0.05

In *P. falciparum* infections, individuals over 25 years old were less likely to carry gametocytes than children under 5 years old (a*OR* = 0.16, 95% *CI:* 0.03–0.85, *P* = 0.0257) (Table 3). Similar to *P. vivax*, *P. falciparum* malaria patients with a history of malaria had significantly higher odds of gametocyte positivity compared to those without previous malaria history (a*OR* = 66.76, 95% *CI:* 27.17–180.31, *P* < 0.001) (Table 3). Interestingly, parasite density was identified as a protective factor against gametocyte carriage. Patients with high falciparum parasite density had lower odds of gametocyte positivity compared to those with low parasite density (a*OR* = 0.85, 95% *CI:* 0.73–0.99, *P* = 0.0358) (Table 3).

### Risk factors for *Plasmodium* gametocyte density among gametocyte carriers

The results of the multivariable analysis for gametocyte density identified several statistically significant factors influencing gametocyte density in *P. vivax* infections. Similar to the risk factors for gametocyte carriage, seasonal factors, age, and parasite density had a significant impact, with a lower gametocyte density during the rainy season compared to the dry season (a*OR* = 0.70, 95% *CI:* 0.66–0.75, *P* < 0.001) (Table 4). Furthermore, compared to children under 5 years old, individuals over 25 years old had lower gametocyte density (a*OR* = 0.84, 95% *CI:* 0.76–0.93, *P* = 0.0012) (Table 4). Patients with high parasite density exhibited higher gametocyte density compared to those with low parasite density (a*OR* = 1.09, 95% *CI:* 1.06–1.11, *P* < 0.001) (Table 4). Similarly, travel history also had a statistically significant effect; individuals with a travel history had more than twice the gametocyte density (a*OR* = 2.07, 95% *CI:* 1.48–2.91, *P* < 0.001) (Table 4). However, having a previous episode of malaria within the last 12 months was associated with lower gametocyte density compared to individuals without a history of malaria (a*OR* = 0.53, 95% *CI:* 0.49–0.57, *P* < 0.001) (Table 4). Body temperature was also a protective factor for gametocyte density in *P. vivax*. Patients with fever had lower gametocyte density compared to those with normal body temperature (a*OR* = 0.86, 95% *CI:* 0.81–0.92, *P* < 0.001) (Table 4). There was also a difference in gametocyte density by sex. Compared to males, females had lower gametocyte density (a*OR* = 0.88, 95% *CI:* 0.83–0.93, *P* < 0.001) (Table 4). Furthermore, gametocyte density decreased annually (a*OR* = 0.78, 95% *CI:* 0.77–0.79, *P* < 0.001) (Table 4).

**Table 4:** Results from multivariable Gaussian regression for risk factors for density in *Plasmodium vivax* and *P. falciparum* infections

Variables	*P. vivax*		*P. falciparum*	
	a*OR* (95% *CI*)	*P-*value	a*OR* (95% *CI*)	*P-*value
Season				
Dry	1		1	
Rainy (May to October)	0.70 (0.66–0.75)	** < 0.001**	0.14 (0.03–0.60)	**0.0115**
Sex				
Male	1		1	
Female	0.88 (0.83–0.93)	** < 0.001**	1.73 (0.40–7.54)	0.4695
Ethnicity				
Non-Kachin	1		1	
Kachin	1.05 (0.90–1.23)	0.5435	0.18 (0.00–38.69)	0.5372
Age (years)				
< 5	1		1	
5–14	0.93 (0.84–1.02)	0.1314	2.05 (0.29–14.2)	0.4732
15–24	0.95 (0.86–1.05)	0.3037	0.94 (0.15–5.88)	0.9494
25 +	0.84 (0.76–0.93)	**0.0012**	0.76 (0.10–5.76)	0.7907
Malaria history^a^				
No	1		1	
Yes	0.53 (0.49–0.57)	** < 0.001**	0.84 (0.25–2.83)	0.7860
Travel history				
No	1		1	
Yes	2.07 (1.48–2.91)	** < 0.001**	0.53 (0.01–25.67)	0.7493
Fever				
No	1		1	
Yes	0.86 (0.81–0.92)	** < 0.001**	1.28 (0.38–4.28)	0.6948
Parasite density (Log, parasites/μl)	1.09 (1.06–1.11)	** < 0.001**	1.01 (0.81–1.25)	0.9349
Year	0.78 (0.77–0.79)	** < 0.001**	1.08 (0.79–1.48)	0.6121

a*OR* adjusted odds ratios, *CI* confidence interval

^a^Malaria reported in the past 12 months

Bold indicates a *P* value less than 0.05

In *P. falciparum* gametocyte carriers, gametocyte density was lower in the rainy season compared to the dry season (a*OR* = 0.14, 95% *CI:* 0.03–0.60, *P* = 0.0115) (Table 4). Unlike *P. vivax*, no significant differences were observed in body temperature (a*OR* = 1.28, 95% *CI*: 0.38–4.28, *P* = 0.6948), sex (a*OR* = 1.73, 95% *CI:* 0.40–7.54, *P* = 0.4695), malaria history (a*OR* = 0.84, 95% *CI:* 0.25–2.83, *P* = 0.7860), travel history (a*OR* = 0.53, 95% *CI:* 0.01–25,67 *P* = 0.7493), and asexual parasite density (a*OR* = 1.01, 95% *CI:* 0.81–1.25, *P* = 0.9349) (Table 4).

### Effect of genotyping on *Plasmodium* gametocyte carriage and gametocytemia

**Fig. 3:**
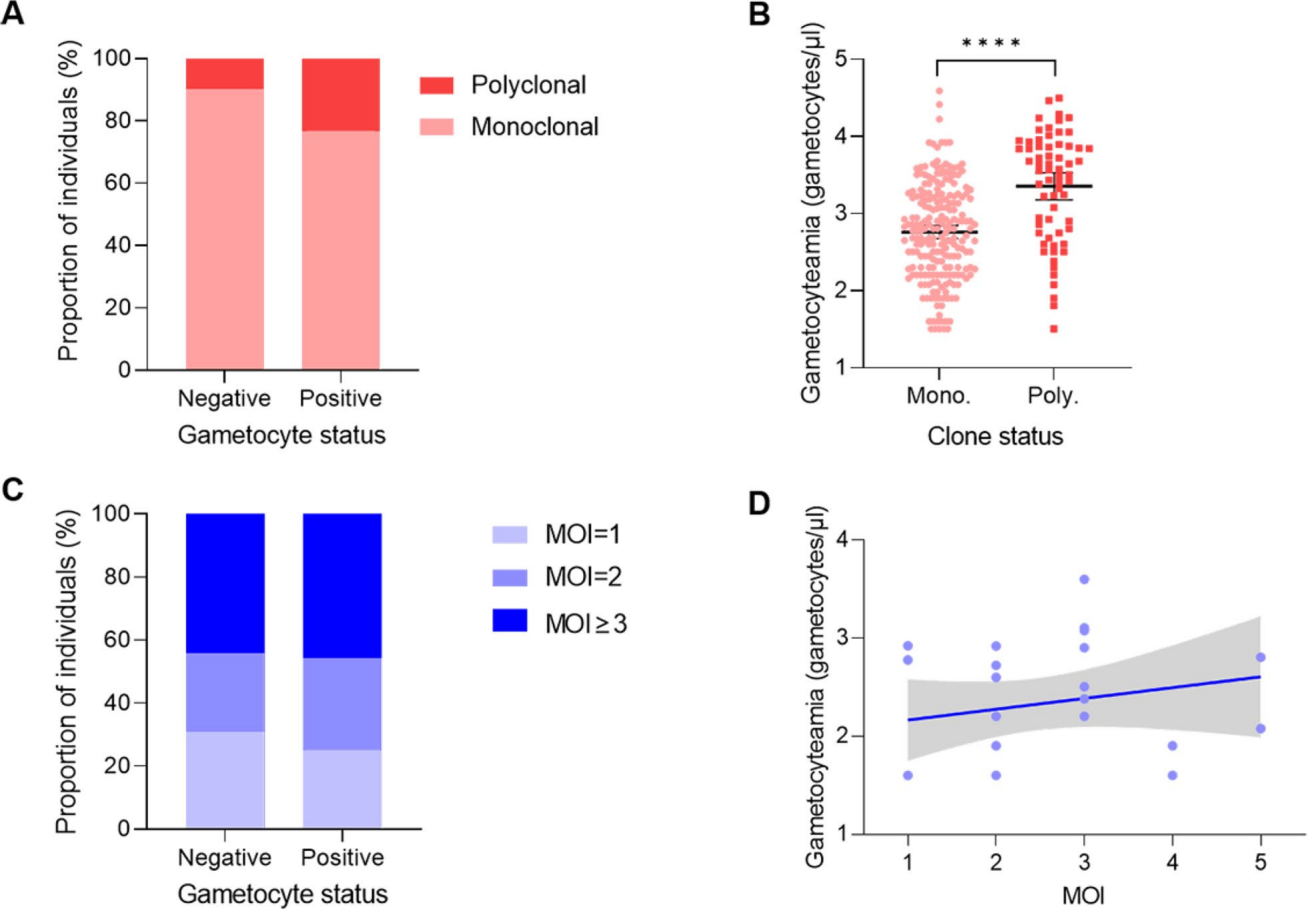
Relationship between gametocyte carriage or gametocytemia (gametocytes/µl of blood) and MOI value in *Plasmodium vivax* (**A** and **B**) and *P. falciparum* (**C** and **D**). Gametocytemia on the y-axis is on a log10 scale. *MOI* Multiplicity of infection

Of the 339 randomly selected *P. vivax* samples, 331 were successfully genotyped. Three main *Pvmsp-3α* genotypes were identified based on their PCR product size: type A (1900–2000 bp), type B (1400–1500 bp), and type C (1100–1300 bp). RFLP analysis revealed a total of 19 cases of mixed infection. The PCR products of *Pvmsp-3β* showed two main types of size polymorphism based on PCR product size: type A (1700–2200 bp) and type B (1400–1500 bp). RFLP fragments digested by *Pst*I showed a total of 59 cases of mixed infection. Combining *Pvmsp-3α* and *Pvmsp-3β* genes, PCR–RFLP analysis detected a total of 70 mixed infection cases. In *P. vivax* populations, the proportion of polyclonal infection in samples with gametocyte carriers was significantly higher than in samples without gametocytes (*P* = 0.0388, Fig. 3A). The mean *P. vivax* gametocyte density in polyclonal infections was significantly higher than in monoclonal infections (*P* < 0.0001, Fig. 3B).

Although 510 *P. falciparum* malaria samples were examined by microscopy, only 166 DBSs were collected from these clinical cases. Among these, 24 samples were diagnosed as gametocyte-positive by light microscopy. All DBS samples of *P. falciparum* were successfully genotyped, with the MAD20 allele gene family being the most common *Pfmsp1* gene variation in infected patients, while the 3D7 allele gene family in *Pfmsp2* was more prevalent than the FC27 family. The MOI range was 1–5, and the proportion of infections with MOI ≥ 2 tended to be higher in samples with gametocyte carriers than in samples without gametocytes (*P* > 0.05, Fig. 3C). *P. falciparum* gametocyte density increased with the MOI value (*P* > 0.05, Fig. 3D).

## Discussion

Efforts to control malaria along international borders are crucial for achieving malaria elimination. Despite strengthened malaria control measures, such as the widespread use of long-lasting insecticidal nets (LLINs) and indoor residual spraying (IRS), a significant malaria outbreak occurred between 2015 and 2017, and a rebound occurred in 2020 along the China-Myanmar border. Notably, the outbreak of COVID-19 in 2019 likely disrupted effective malaria control measures by diverting resources and attention from malaria-related activities, potentially contributing to an increase in malaria cases [29, 30]. Gametocytes play a critical role in the life cycle of malaria parasites and serve as a key link in the transmission of malaria from humans to mosquitoes. A deeper understanding of gametocyte carriage dynamics and their transmission implications can inform more effective, targeted interventions to reduce the infectious reservoir and achieve malaria elimination.

During the 10-year study (2011–2020), gametocyte carriage occurred more frequently during the rainy season, following the same general pattern as symptomatic malaria infections (although the proportion of gametocyte-positive individuals was highest during the dry season, when the overall number of cases was lower). This finding is consistent with prior reports indicating that the prevalence of gametocytes in other transmission settings generally correlates with the prevalence of asexual parasites [31, 32]. Consistent with earlier studies, gametocytes were observed more frequently in *P. vivax* infections than in *P. falciparum* infections [33]. This may be attributed to the distinct biological characteristics of these two parasite species. *P. vivax* gametocytes mature faster and can be detected in the bloodstream within 3 to 5 days after the initial microscopic identification of asexually replicating parasites. In contrast, *P. falciparum* gametocytes exhibit markedly different morphological features, require 8 to 10 days to mature, and accumulate and sequester during their development in the bone marrow parenchyma before emerging into the blood circulation [34, 35]. Nevertheless, gametocyte carriage and density can be influenced by various other factors, including individual or host characteristics, geographical epidemiological transmission patterns, human host factors, parasite biology, and intervention strategies [36–41].

Environmental factors, including climate, temperature, humidity, and seasonality, may influence the prevalence of gametocytes. In certain tropical regions with endemic malaria, transmission predominantly occurs during the rainy season, which typically lasts several months, whereas malaria transmission ceases during the prolonged dry season [42]. Conversely, some studies in areas with high malaria transmission intensity have indicated that seasonality has minimal or no impact on the proportion of gametocyte-positive infections [43]. In Thailand, research has demonstrated that the prevalence of gametocyte in both *P. vivax* and *P. falciparum* infections is significantly higher during the dry season compared to the rainy season, showing a negative correlation with rainfall levels [44, 45]. Consistent with these findings, this study observed a significant increase in the proportion of gametocyte positivity for both *P. vivax* and *P. falciparum* during the dry season relative to the rainy season. Although the number of mosquitoes is often lower in the dry season compared to the rainy season, mosquito activity becomes more concentrated and efficient, thereby enhancing the likelihood of malaria transmission [46, 47]. Relatedly, a second peak of malaria cases also occurs in some parts of the GMS, although the seasonal peak during the rainy season is most consistent in the malarious regions of this area [48].

Our results indicate that older age in children and adults serves as a protective factor against gametocyte carriage in both *P. vivax* and *P. falciparum*, correlating with lower gametocytemia levels in *P. vivax*. Similar patterns of gametocyte carriage have been reported in other studies, where gametocyte prevalence was higher among the younger age groups [36, 43, 49, 50]. With increasing age, factors such as the maturation of the immune system, the formation of immune memory, enhanced antibody levels, and immune tolerance developed through long-term infections may gradually improve the host’s ability to control malaria parasites [36]. The high prevalence of gametocyte carriage among the younger age group (< 5 years) highlights their potential role in sustaining malaria transmission in the region. Children have been identified as critical contributors to the malaria infectious reservoir in various settings [36]. However, some studies report that adults can exhibit significantly higher gametocytemia compared to children under the age of 12 years [36, 51]. This may also be attributed to their behavior, body size and relative exposure to mosquito bites [22].

Patients with a history of malaria infection constitute a substantial risk factor for *Plasmodium* gametocyte carriage in this study. This observation is consistent with a 19-year longitudinal analysis of a malaria cohort, which revealed that previous episodes of clinical malaria contribute to the development of gametocyte carriage [52]. Previous malaria infections significantly increase the likelihood of gametocyte carriage due to the complex interplay of biological, immunological, and epidemiological factors. In *P. vivax*, many episodes arise from hypnozoite stages in the liver that remain dormant following previous infections via an unknown mechanism. The presence of hypnozoites leads to relapses, causing recurrent waves of asexual parasites and gametocytes [53]. Even in individuals with partial immunity, these relapses contribute to sustained gametocyte carriage. In *P. falciparum*, chronic infections and immune pressure can lead to low-level, ongoing gametocyte production [54]. An epidemiological study conducted along the western border of Thailand indicates that recrudescent infections increase the risk of persistent gametocyte carriage in *P. falciparum* by 2.3 times [55]. Furthermore, our study indicates that a history of malaria infection may paradoxically lead to a reduction in gametocyte density in *P. vivax*. Antibodies targeting malaria parasites are produced in individuals with a history of malaria infection, including those that respond to gametocyte-infected erythrocyte surface antigens during the human blood cycle, potentially reducing gametocytemia in these patients [56–58].

Moreover, we found that fever exerts a positive influence on gametocyte prevalence but is negatively associated with gametocytemia in *P. vivax* infections*.* Consistently, McKenzie et al. reported that patients with gametocytes had higher fever and parasitemia than those without gametocytes in *P. vivax* infections, suggesting that the presence of gametocytes may exacerbate febrile responses [33, 59]. In contrast, a study in Gambian indicated that children with fever (> 37.4 ℃) or high parasite densities (> 100,000 parasites/ml) were less likely to carry gametocytes [60]. This discrepancy may reflect regional differences in malaria transmission dynamics or could be related to variations in patient immunity, such as inflammatory responses. Additionally, travel history was associated with increased gametocyte density in *P. vivax* infections in this study. Some studies have reported that individuals with a history of travel to malaria-endemic areas have a higher risk of malaria infection, particularly in regions with relatively high transmission intensity [61], although no direct correlation with gametocyte density has been established.

Regarding parasitic factors, it is crucial to note that *P. falciparum* and *P. vivax* exhibit different patterns of gametocyte development and transmission. In *P. falciparum*, gametocytes typically appear 10–14 days post-infection, whereas in *P. vivax*, gametocytes emerge concurrently with asexual stages [62]. Furthermore, parasite density and the stage of infection can significantly influence gametocyte carriage.For example, asexual parasitemia is associated with gametocyte carriage in both *P. falciparum* and *P. vivax* [63–66], and higher asexual parasitemia levels are linked to an increased risk of gametocytemia [67]. Our findings indicate that asexual parasite density serves as a risk factor for both gametocyte carriage and density in *P. vivax,* but acts as a protective factor against gametocyte production in *P. falciparum*.

Previous studies have demonstrated that mixed-genotype infections lead to increased transmission compared to single-clone infections in rodent *Plasmodium* infections [68]. Additionally, it has been reported that MOI can lead to prolonged or persistent gametocyte production [19, 69]. It has been proposed that polyclonal *P. falciparum* infections last longer than monoclonal infections, and can increase the likelihood of gametocyte production [70], although some studies have found no correlation between genetic diversity and gametocyte prevalence [71, 72]. In this study, we found that *P. falciparum*-infected patients with gametocytes exhibited relatively high polyclonality, suggesting that intraspecific competition within *Plasmodium* infections may facilitate gametocyte development in this population. We also found that polyclonal infections are significantly correlated with gametocyte carriage and density in *P. vivax*. These polyclonal infections may persist in individuals, enhancing gametocyte carriage and providing increased opportunities for certain parasite variants to evade the host immune system’s antiparasitic response while dedicating resources to gametocyte formation [70], thereby promoting both the development and persistence of gametocytes.

Despite the significant findings of this study, several limitations should be acknowledged. First, while molecular tools (e.g., qRT-PCR targeting *pvs25*/*pfs25*) offer substantially greater sensitivity for gametocyte detection, logistical constraints in this resource-limited setting necessitated reliance on LM [73, 74]. The evidence demonstrates that gametocyte carriage is nearly universal in both *P. vivax* and *P. falciparum* infections when assessed by molecular methods, with frequently submicroscopic yet remaining infectious to mosquitoes [75]. Therefore, our LM-derived estimates of gametocyte prevalence and density likely represent a lower bound, with true carriage rates potentially exceeding those reported. Importantly, LM remains clinically relevant as it effectively identifies the most infectious individuals—those with gametocytemia levels strongly predictive of mosquito infectivity [12]. Second, although our genotyping approach reliable identifies predominant clones, it exhibits reduced sensitivity in detecting polyclonal infection and minority clones compared to recent amplicon deep sequencing methods [76, 77]. Additionally, while our PCR–RFLP based method offers estimates of clonal diversity, employing a maximum likelihood approach with COIL using the initially targeted SNPs would allow for more precise estimation of the probable number of clones in each *P. vivax* infection [78, 79]. The sample size of *P. falciparum* was relatively small, which may limit the generalizability of the results. However, our findings here do reflect the ongoing reduction of *P. falciparum* transmission along the China-Myanmar border. Third, accurate treatment information was not collected for many patients with a history of malaria infection, as specific information on treatment regimens and durations was unavailable. Previous studies have shown that treatment with conventional antimalarial drugs such as chloroquine, amodiaquine, sulfadoxine-pyrimethamine, or their combinations is associated with persistence of *P. falciparum* gametocytemia for weeks to months [80]. Additionally, observations from malariotherapy patients suggest that co-infection with *P. malariae* may increase *P. falciparum* gametocyte production [81]. A recent short communication indicates that co-infection can either enhance or decrease gametocyte carriage, depending on the specific interactions between the species involved [82]. Due to the limited number of microscopic gametocyte-positive cases for *P. falciparum*, the effect of co-infection on gametocyte production was not analyzed. Finally, this study focused on symptomatic patients and would undoubtedly have missed other infections in the broader communities from which these patients originate (including symptomatic patients who do not seek treatment and individuals who are asymptomatic and therefore have no suspected reason to seek care). While clinical cases remain a priority for treatment and surveillance, their contribution to overall transmission may be eclipsed by asymptomatic reservoirs in elimination settings, as asymptomatic infections frequently harbor persistent infectious gametocytes [83, 84]. Our analysis should therefore not necessarily be considered to be representative of the entire parasite reservoir in this setting or elsewhere. Nevertheless, this study provides valuable insights into potential factors affecting gametocyte carriage and density, contributing to a deeper understanding of the risk factors related to malaria gametocyte along the China-Myanmar border.

## Conclusions

This study highlights the complex interplay of biological, environmental, and epidemiological factors that influence malaria gametocyte carriage and density along the China-Myanmar border. Younger age, a history of malaria infection, travel, and polyclonal infections emerged as key contributors to increased gametocyte prevalence, particularly in *P. vivax* infections. Gametocyte carriage and density were higher in the dry season than in the rainy season for both species (*P. falciparum* and *P. vivax*). These findings underscore the importance of targeted strategies that consider host, parasite, and environmental dynamics to effectively reduce the infectious reservoir and advance malaria elimination efforts in border regions.

## Data Availability

All data analyzed for this study are included within the article.

## Abbreviations

COVID-19: Coronavirus disease 2019
GMS: Greater Mekong subregion
MOI: Multiplicity of infection
*Pfmsp1*: *Plasmodium falciparum* Merozoite surface protein 1
*Pfmsp2*: *Plasmodium falciparum* Merozoite surface protein 2
*Pvmsp-3α*: *Plasmodium vivax* Merozoite surface protein 3α
*Pvmsp-3β*: *Plasmodium vivax* Merozoite surface protein 3β
ICEMR: International Center of Excellence for Malaria Research
PCD: Passive case detection
IDP: Internally displaced people
DBS: Dried blood spot
LM: Light microscope
WBC: White blood cell
gDNA: Genomic DNA
PCR–RFLP: Polymerase chain reaction-restriction fragment length polymorphism
LLR: Log likelihood ratio
GLM: Generalized linear model
a*OR*: Adjusted odds ratio
*CI*: Confidence intervals
SI: Seasonal index
LLIN: Long-lasting insecticidal net
IRS: Long-lasting insecticidal net
